# Community perceptions, attitude, practices and treatment seeking behaviour for schistosomiasis in L. Victoria islands in Uganda

**DOI:** 10.1186/1756-0500-7-900

**Published:** 2014-12-11

**Authors:** Narcis Kabatereine, Fiona Fleming, Wangechi Thuo, Benjamin Tinkitina, Edridah M Tukahebwa, Alan Fenwick

**Affiliations:** Schistosomiasis Control Initiative of Imperial College London, London, UK; Vector Control Division, Ministry of Health Uganda, Kampala, Uganda; The Global Network for Neglected Tropical Diseases, Washington, D.C, USA

## Abstract

**Background:**

Over 200,000 people, most of them infected with *Schistosoma mansoni* inhabit 150 islands in Lake Victoria in Uganda. Although a programme to control the disease has been ongoing since 2003, its implementation in islands is inadequate due to high transport costs on water. In 2011 and 2012, the Global Network for Neglected Tropical Diseases (GNNTD) through Schistosomiasis Control Initiative (SCI) provided financial support to ease treatment delivery on the islands and over the period, therapeutic coverage has been increasing. We conducted a study with an objective to assess community awareness of existence of the disease, its signs, symptoms, causes and transmission as well as attitude, practice and health seeking behavior.

**Methods:**

This was a cross sectional descriptive study which used pre-tested interviewer administered questionnaire among purposively selected individuals in schools, health facilities and communities. Frequency distribution tables, graphs and cross tabulations were the main forms of data presentation.

**Results:**

Our results showed that there are numerous challenges that must be overcome to achieve effective control of schistosomiasis in the islands. Many people especially young men are constantly on the move from island to island in search for richer fishing grounds and such groups are commonly known to miss treatment by mass chemotherapy. Unfortunately case management in health facilities is very poor; health facilities are few and understaffed mainly with unskilled personnel who are overburdened by other illnesses such as malaria and HIV and the supply of praziquantel in health facilities is inadequate. Furthermore, sanitation is appalling, no clean water and community knowledge about schistosomiasis is low even among biomedical staff.

**Conclusion:**

Rather than elimination, our results indicate that the programme should continue to target morbidity control beyond the 2020s until preventive measures have been instituted. The government should provide adequate trained health workers and stock praziquantel in all island health facilities.

## Background

Schistosomiasis (bilharzia) is a water-borne parasitic infection, caused by six species of blood flukes of genus *Schistosoma*[1]. It is an infectious disease that affects more than 230 million people worldwide [2], causing an estimated 3.3 million disability- adjusted years (DALYs), [3]. It occurs mainly among rural dwellers in tropical and subtropical countries where poverty, inadequate sanitation and poor health awareness favour the disease transmission [4]. Globally, it is found in 74 countries with Sub Saharan Africa accounting for 93% of the cases [5]. Over the last fifty years, schistosomiasis distribution has changed a little; reduced in some areas due to successful control but increased in others due to population growth and increased water development projects.

In Uganda, intestinal schistosomiasis caused by *Schistosoma mansoni* is the most common type and has been known for a long time on shores of Lakes Albert, Victoria and Kyoga and along the Albert Nile [6–8]. The disease occurs in 63 out of 112 districts but urinary schistosomiasis due to *S. haematobium* has been reported in only two of these districts [9]. It is estimated that four million people are infected while 16.7 million are at risk of the infection [9].

Despite its serious health consequences and anchoring large numbers of people in poverty, schistosomiasis has been largely neglected over the years especially in Sub Saharan Africa perhaps because its mortality rate is considered low compared with many other infectious diseases. However, over the past decade, effort to control the disease has been scaled up [10] following World Health Assembly resolution 54.19 encouraging endemic countries to annually deworm at least 75% of school aged children and high risk communities [11]. By 2009, up to 21 out of 76 endemic countries had initiated their control programmes [12].

Having been encouraged by significant success in some countries, and with increased donation of praziquantel, the 65th World Health Assembly (WHA) recommended that countries should scale up treatment coverage and where feasible they should target elimination through preventive chemotherapy as the mainstay [13], but with supportive measures including health education, provision of safe water and sanitation improvement [12].

In Uganda, schistosomiasis control began in the early 1990s but it was insubstantial. It was boosted by financial support from the Schistosomiasis Control Initiative (SCI) in 2003 [14] targeting morbidity control through annual mass treatment [15]. By 2006, the prevalence and intensity of infection in majority of the foci had been drastically reduced [16] and transmission interruption seemed potentially possible in majority of the affected areas. The United States Agency for International Development (USAID) through Research Triangle Institute (RTI) expanded the interventions by launching an integrated NTD control programme in 2007 targeting LF, schistosomiasis, trachoma, Onchocerciasis, and STH [17].

However, due to high transport costs on water, operations in the islands were expensive, only 3 out of 150 inhabited islands had been mapped [18] and hence both geographical and chemotherapeutic coverage were extremely low. Mapping in the rest of islands was completed in 2010 with financial support from the Global Network for Neglected Tropical Diseases (GNNTD). The results showed that all the 150 islands qualified for annual mass treatment which was initiated immediately achieving a therapeutic coverage of 69% [18] and 74.1% in 2012 (Vector Control Division (VCD), unpublished). Unfortunately, little attention was made to promote preventive measures and hence reinfection rate remained very high [19, 20]. Disease transmission was also favored by unstable nature of the island population who included migrants from schistosomiasis endemic regions [18]. Most islanders do not own land, they live in temporary low quality houses with poor sanitation, lack of clean water, and they are overcrowd and all these factors favour schistosomiasis transmission [18].

In this paper, we report the findings of a study to assess community awareness of existence of schistosomiasis, its signs, symptoms, causes and transmission factors. Also assessed were peoples’ attitude, practice, health seeking behaviour and skills for schistosomiasis case management. The availability of safe water and sanitary facilities and the extent to which they are utilized were also examined. Since school based treatment is the major approach towards schistosomiasis control in the country [14], it was feared that school attendance might be low and that the approach may not work in the islands [21]. Therefore, emphasis was made to determine the island school enrolment rate. The collected data will be utilised in designing complementary preventive intervention messages and will also serve as reference for evaluating the impact of the current control measures.

## Methods

### Study area and population

The study took place in 9 districts that surround Lake Victoria in Uganda namely Mayuge, Jinja, Buikwe, Buvuma, Mukono, Wakiso, Mpigi, Masaka and Kalangala. Administratively, the districts are sub-divided into sub-counties, parishes and finally villages. Altogether, there are 212 islands but the study was limited to 150 inhabited islands with a total population of 220,736 people [18].

### Study design

This was a cross sectional descriptive Knowledge, Attitude and Practice (KAP) study which used pre-tested interviewer administered questionnaire among purposively selected individuals in schools, health facilities and communities. The questionnaire focused on schistosomiasis and its control”, coupled with collection of geographical coordinates (Garmin GPSMAP 62, Garmin Ltd, Southhampton, UK) of all households, health facilities and schools visited. After the pre-test, the questionnaire was perfected, interviewers trained and the questionnaire was administered immediately following initiation of MDA in each village. To avoid the potential for interviewer bias, a single trained person administered the questionnaire in each district.

### School and community questionnaire

All schools in a village were involved in the study and one teacher and two pupils were interviewed for each school. In communities, randomly selected household heads were interviewed. The randomization was done by selecting the third household on the left hand side of the school main gate and thereafter every tenth household in that direction. If there was no school in that village, the entrance of the chairman’s residence was used for the same purpose. If there was no adult in a selected household, the next household was visited until a person aged at least 25 years was located.

The questions included disease signs and symptoms and preventive measures. Other questions were on impact of helminth infections, perceived benefits of treatment, treatment seeking behavior and praziquantel side effects. Also interviewed were Community Medicine Distributors (CMDs) on their role in the delivery of treatment, their willingness to continue with that job and on their incentives.

### Health workers questionnaire

In health centers, only the officer in-charge of the facility or acting as facility head was interviewed mainly on diagnostic equipment and management of cases. Information was gathered about the qualifications of biomedical staff, medicines dispensed and on knowledge, perceptions and practice of health workers regarding causes, symptoms and preventive measures. Thereafter, all health workers in a facility were sensitized about approaches to good case management. A full copy of the main questions is shown in annex.

### Data analysis

The collected data was double entered in a computer by two data clerks using Epi-Info 3.0 (Centers for Disease Control and Prevention, Atlanta, GA, USA)’. It was cleaned and then exported into Stata® 10 for analysis. Since the study was descriptive, frequency distribution tables, graphs and cross tabulations were the main forms of data presentation. One was considered knowledgeable about schistosomiasis if he/she correctly answered three quarters of questions in this area. Odds ratios were estimated in the household data using logistic regression to identify variables associated with knowledge. None overlapping 95% confidence intervals and p values <0.05 were considered as significance levels. GPS coordinates were transferred to Health Mapper® 4.3.2 software (http://www.healthmap.org/en/) to generate distribution maps of schools, health facilities and households visited.

### Ethical considerations

Ethical clearance for the study was obtained from “Vector Control Division Ethical Committee” and the Ugandan National Council of Science and Technology. A flyer explaining the study procedure was provided to each participant, head teachers and village political leaders before questionnaire administration. Written informed consent was obtained from health worker, community medicine distributors and teachers. Furthermore, a verbal consent was taken from parent/guardians of school children. All those who chose to join the study were informed that they had a right to refuse or to withdraw their participation. At the end of the survey, all villages including schools and any other institutions in the islands were offered free praziquantel (Distocide 600 mg, Shin Poong Pharmaceuticals, Seoul Republic of Korea, 40 mg/kg body weight) against schistosomiasis and free albendazole against soil transmitted helminthiasis (STH) in the frame of the normal mass drug administration (MDA) [22].

## Results

### Description of study participants

**Figure 1:**
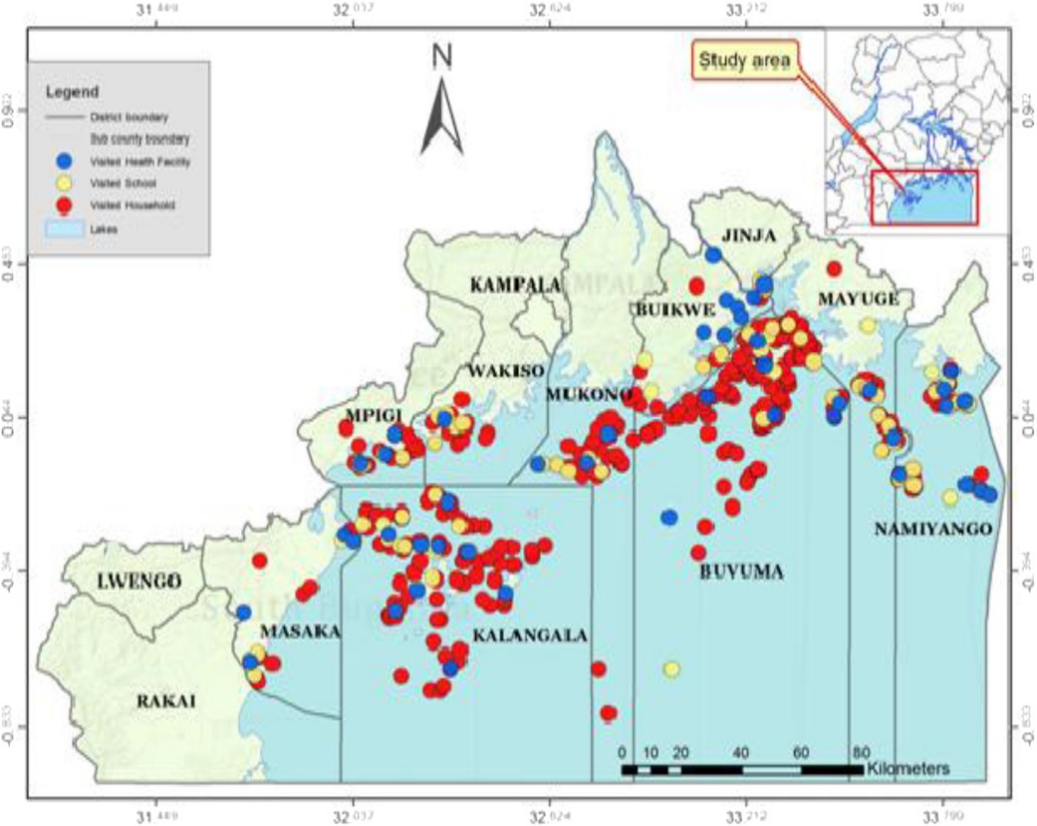
**To show the distribution of health facilities, schools and households visited during the survey.** Generated by Health Mapper® 4.3.2 software (http://www.healthmap.org/en/).

Altogether, 908 household heads, 286 drug distributors, 181 pupils, 104 teachers and 47 biomedical health workers participated in the study and the distribution of the visited households, schools and health facilities are shown in Figure 1.

Of the 908 household heads, 466 (51.3%) were males and 442 (48.7%) were females with an average age of 35.7 years and about half of them were illiterate or semiliterate. Their economic activities included; trade mainly in fish (30.4%), fishing (29.4%) and peasant farmers (29.3%) in that order. Of these, 64% (578) had lived in their villages for at least 5 years and 54% (487) were permanent island dwellers. On average, there were 2 school age children (5–15 years) per household and most of them (83.3%, 1682/2020) were enrolled in school (Table 1).

**Table 1:** **Distribution and demographic characteristics of respondents in community questionnaire survey**

Variables	No of respondents	Percentage (%)
Sex		
Female	442	48.7
Male	466	51.3
Main economic activity*		
Business (mainly in fish)	276	30.4
Fishing	267	29.4
Farming	266	29.3
Civil servants	23	2.5
Education level		
None	127	14.0
Primary	471	51.9
Secondary	269	29.6
Tertiary	41	4.5
How long have you lived in this village		
<2 years	188	20.7
2 < 5 years	140	15.4
≥5 < 10 years	209	23.0
10 + years	369	40.6
Do you sometimes live islands to mainland for more than a month?		
No	487	54.7
Yes	404	45.3

*Multiple responses were considered.

A total of 285 people including 104 teachers and 181 pupils responded to the school questionnaire. The mean school enrolment was 295 pupils and on average, 49.8% were females and 88.5% of the schools were located within 5 kilometers from the Lake shore, the suspected source of transmission. The major source of drinking water in schools was a borehole at 35.8% and lake water at 34.4%. Of the 47 health facilities, 55% (26/47) were Health Centre IIs (HC II), the lowest health facility in Uganda. Overall, the total biomedical staff in islands was 271 of whom 33.9% (92) were nursing assistants (trained on the job), 19.2% (52) enrolled nurses, 12.5% (34) clinical officers and 3.7% (10) medical officers (doctors). However, there was no hospital in islands. Only one of the island health facilities received as many as 100 clients a day while the rest served between 20 and 99 clients. Most facilities (61.7%) had a single staff and doctors were present in just 4/47 facilities.

### Facility based management of schistosomiasis cases

Although 57% of health facilities had a microscope, only 39% (11) had a working laboratory implying that even some of those with a microscope had no functional laboratory perhaps due to lack of skilled staff. For diagnosis of cases, 75% (21) of the facilities relied on clerking for symptoms the commonest of which were diarrhea (46.8%), abdominal pain (44.7%), enlarged stomach (23.4%), blood in stool (14.9%), fever (4.5%) and anemia (2.1%) (Table 2) but just 69% (28) offered case management perhaps due to lack of PZQ or due to limited knowledge about the disease.

**Table 2:** **To show response to questions regarding management of schistosomiasis cases in health facilities**

Variables	No of respondents	%
Is bilharzia one of the diseases treated in this health facility (n = 47)		
No	19	40.4
Yes	28	59.6
How do you determine whom to treat (n = 28)		
Laboratory results	11	39.3
Symptoms	21	75
Is PZQ dispensed in the health facility (n = 28)?		
Yes	0	0
No	28	100
Can these drugs be purchased in the vicinity? (n = 47)		
Yes	24	54.5
No	23	45.5

### Access and utilization of health facilities among household participants

About 36.5% of household heads lived within five kilometers from the nearest health facility and 55% had utilized facility services at least once within the last six months. Of those who had never visited a facility, 58% claimed that the distance was long, 29.3% lacked funds for transport and that often facilities lacked medicine (34.1%). In spite of these limitations, 80.1% of household heads asserted that if they suffered from schistosomiasis, they would go to a health facility for remedy and only 21% opted to go to a drug distributor while none sought treatment from a traditional healer. Females visited significantly more often (59.5%) than males (52.3%), p = 0.014. Level of education did not impact on treatment seeking behavior (Table 3).

**Table 3:** **To show proportion of respondents who sought treatment from health facilities by sex, education and distance from nearest health facility**

Variables	Total respondents	Number times sought treatment from health facilities in last 6 months (%)				
		None	Once	Twice	3 + times	P
Sex						
Female	442	179(40.5)	81(18.3)	59(13.3)	123(27.8)	
Male	466	227(48.7)	90(19.3)	59(12.7)	90(19.3)	0.01
Education						
None	127	58(45.7)	19(15.0)	16(12.6)	34(26.8)	
Primary	471	215(45.6)	81(17.2)	66(14.0)	109(23.1)	
Secondary	269	108(40.1)	67(24.9)	31(11.5)	63(23.4)	
Tertiary	41	25(61.0)	4(9.8)	5(12.2)	7(17.1)	0.09

### Sanitation in schools and communities

**Figure 2:**
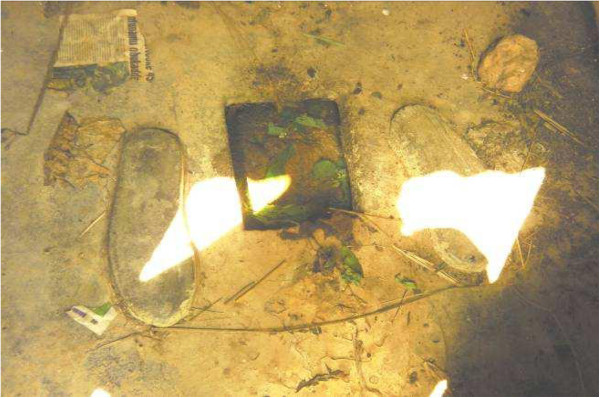
**To show that majority of island latrines were full and dirty and thus not convenient for use.**

**Figure 3:**
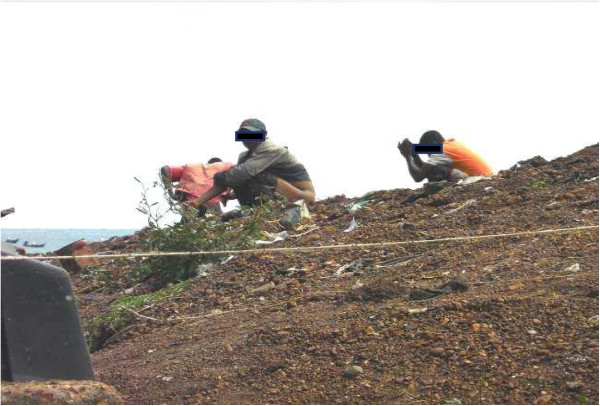
**To show that most people in Islands practiced open air defaecation (Taken using a zoom camera).**

All schools had latrines and 77.5% of school respondents used them. Most latrines had less than 10 stances separated equally by gender. However, observations of the latrines showed that 15% were extremely filthy, 10.5% had no doors and 13.7% were either full (Figure 2) or almost collapsed implying that most latrines were not convenient for use. Although 90.8% of household heads believed everyone should use a latrine, only 33% had a latrine at home. Follow-up observations around the concerned homes showed that majority of the available latrines were either communal or shared between many families. These latrines were poorly constructed, dilapidated and dirty and with stool scattered in their surroundings. Thus open air defaecation was common in all islands (Figure 3). Only Wakiso and Mpigi districts had average latrine coverage of 63% and 50% respectively (Table 4) while the coverage was less than 50% in all other districts lowest being in Jinja and Buvuma islands at 11% each (Table 4).

**Table 4:** **To show latrine coverage and hand washing facilities in island schools and households by district**

District	Households		School		
	Total number	n (%) with a latrine	Total number	n (%) with washing facility	Hand
Buikwe	28	6 (21.4)	6	2 (33.3)	
Buvuma	254	29 (11.4)	15	3 (20)	
Jinja	9	1 (11.1)	3	0 (0.0)	
Kalangala	196	83 (42.3)	18	11 (61.1)	
Masaka	24	11 (45.8)	7	4 (57.1)	
Mayuge	67	22 (32.8)	9	1 (11.1)	
Mpigi	63	40 (63.5)	7	4 (57.1)	
Mukono	78	23 (29.5)	9	2 (22.2)	
Namayingo	141	68 (48.2)	22	1 (4.5)	
Wakiso	48	24 (50.0)	8	8 (100)	
Over all	908	307 (33.8)	104	36 (34.6)	
P value		<0.001		<0.001	

### Knowledge about schistosomiasis symptoms, mode of transmission and preventive measures by sex, level of education and treatment history

Among biomedical staff, only 38% and 21.3% knew that poor methods of faecal disposal and contact with lake water were associated with schistosomiasis transmission. Drinking dirty water and eating contaminated food were mentioned by (12.8% and 15% respectively as risky while 12.8% had no idea of how transmission occurs. Surprisingly, 52.9% of the household heads knew that one gets schistosomiasis through contact with contaminated water. Unfortunately, fishing and poor sanitary conditions were considered less important at 25.2% and 22.0% respectively. Up to 14.1% of household heads did not know how transmission occurs. 63.8% of biomedical staff thought health education had no role in prevention of infection while 68.1% knew that treatment and latrine use (40.4%) were beneficial. Some household heads (51%) knew that schistosomiasis could be reduced through mass treatment. Nineteen percent and 30% of health workers and household heads respectively thought that the disease could be prevented by drinking boiled water.

Overall, 92.3%, 84.3%, 80.4% and 87.3% of biomedical staff, pupils, teachers and household heads in that order knew schistosomiasis and this difference was insignificant (Table 5). However, among household heads knowledge about the disease was significantly higher among those with tertiary education at 97.6% (CI: 92.6-100.0) compared to those with no education 83.5% (CI: 76.9-90.0) (Table 5). Males were as knowledgeable about the disease as females at 86.9% and 81.4% respectively. Residence status had impact on level of knowledge in that those who had stayed in islands for over 10 years were more informed about the disease at 90% compared to just 72% among those who had been there for less than 2 years. Similar results were observed among school children but not among teachers (Table 5) Knowledge about schistosomiasis was higher among those who had ever been treated compared to those never treated at 100% versus 55.7%), 88.1% versus 57.8% and 98.6% versus 79.8% among household heads, pupils, and teachers in that order (Table 5).

**Table 5:** **To show distribution of knowledge about schistosomiasis among respondents by sex, level of education, residence status, and treatment history**

Variable	Household heads		Pupils		Teachers		HW (n = 47)
	N	% knew bilharzia	N	% knew bilharzia	N	% knew bilharzia	
Overall	908	84.3 (81.9 - 86.6)	179	80.4 (74.6 - 86.3)	104	92.3 (87.1 - 97.5)	87.3 (77.8-96.8)
Sex							
Female	442	81.4 (77.8 - 85.1)					
Male	466	86.9 (83.8 - 90)					
Education							
None	127	83.5 (76.9 - 90)					
Primary	471	82.4 (78.9 - 85.8)					
Secondary	269	85.9 (81.7 - 90.1)					
Tertiary	41	97.6 (92.6 – 100.0)					
Knowledge of bilharzia by length of residence in islands							
<2 years	188	71.8 (65.3 - 78.3)	41	65.9 (50.7 – 81.0)	14	78.6 (54.0 - 100.0)	
2 < 5 years	140	85.7 (79.8 - 91.6)	52	75.0 (62.8 – 87.2)	38	97.4 (92.0 - 100.0)	
5 < 10 years	209	83.7 (78.7 - 88.8)	37	83.8 (71.3 – 96.2)	34	91.2 (81.1 - 100.0)	
10 + years	369	90.2 (87.2 - 93.3)	43	97.7 (93 – 100.0)	16	93.8 (80.4 - 100.0)	
Have you ever been treated for bilharzia							
Yes	585	100 (100–100)	134	88.1 (82.5 – 93.6)	70	98.6 (95.7 - 100.0)	
No	323	55.7 (50.3 - 61.1)	45	57.8 (43.1 – 72.5)	34	79.4 (65.5 – 93.4)	

### Factors affecting knowledge about bilharzia among household respondents

A bivariate analysis of household data revealed that sex, education, residence status, treatment history and age seemed to be associated with knowledge about schistosomiasis (Table 6). For example, males were 1.5 times more likely to know the disease compared to females (p = 0.02). When age was considered, respondents in the age groups 25–44 years and ≥45 years were respectively 2.2 and 2.3 times more likely to know the disease than their counterparts aged 16 – 24 years (Table 6). Although level of education seemed to be associated with knowledge, the association was only significant among those with tertiary education compared to illiterates (p = 0.02). Respondents who had stayed in the islands for 2–4 years, 5–9 years and ≥10 years were 2.4, 2.0 and 3.6 times more likely to know the disease in that order compared to newcomers. In addition, those who thought that everyone should use a latrine were more informed about the disease (OR: 4.2 (2.6-6.9)).

**Table 6:** **To show factors associated with knowledge of schistosomiasis among household respondents**

	Crude odds ratio	
Variable	OR	P
Sex		
Female	1	
Male	1.51 (1.05 - 2.17)	0.02
Age of respondents		
16-24	1	
25-34	1.32 (0.8 - 2.17)	0.28
35-44	2.18 (1.26 - 3.8)	0.01
45+	2.30 (1.2 - 4.41)	0.01
Level of education		
None	1	
Primary	0.93 (0.55 - 1.57)	0.78
Secondary	1.2 (0.67 - 2.15)	0.53
Tertiary	7.92 (0.99 - 63.35)	0.02
Occupation		
Farmer (Y/N)	1.33 (0.88 - 2.02)	0.17
Fisherman (Y/N)	1.23 (0.82 - 1.85)	0.31
Civil servant (Y/N)	1.99 (0.46 - 8.59)	0.35
Merchant (Y/N)	1.2 (0.73 - 1.98)	0.47
How long have you lived in this village		
<2 years	1	
2-4 years	2.36 (1.32 - 4.2)	<0.01
5-9 years	2.02 (1.24 - 3.3)	<0.01
10 + years	3.63 (2.24 - 5.88)	<0.01
Number of times sought treatment from the health facility in the last 6 months		
None	1	
Once	0.99 (0.61 - 1.59)	0.95
Twice	1.62 (0.86 - 3.06)	0.13
3 + times	1.1 (0.7 - 1.73)	0.69
Should everyone use latrine?		
No	1	
Yes	4.2 (2.57 - 6.87)	<0.01

### Perception and attitudes of drug distributors regarding schistosomiasis control

Nearly all medicine distributors (99%) were willing to continue serving. Most of them (88%) had received training and they were satisfied. Those who were not trained were either newly recruited or absent during training. Majority of drug distributors (88%) had been treated at least once. Most villages (88%) had two or more medicine distributors and they believed this number was adequate for the job (78.7%). Severe adverse reactions after MDA were reported by 40.6% of drug distributors and these included diarrhea, vomiting and skin rash at 78.4%, 27.6% and 10.3% in that order.

## Discussion

This study was part of the national mass treatment campaign aimed at identifying how schistosomiasis control interventions can be improved in the islands. Hence almost all potential endemic villages were visited. Our results clearly demonstrate that the island population is extremely unstable. Many people especially young men are on constant move from island to island in search for richer fishing grounds. Asked how long they had resided in the islands, up to 40.6% of household heads had been there for less than 10 years; majority of them for less than 2 years implying that at any one time, most of the islanders are newcomers in the villages where they are found.

Due to their unstable residency, it is difficult to gather large numbers of people for sensitization or treatment; hence many people will inevitably continue to miss treatment during the formal MDA campaigns. In an effort to improve treatment coverage, links were made between the community and the central programme leadership with financial support from GNNTD. Telephone contacts of all relevant island dwellers were collected to keep in touch with them during MDAs [18]. With this approach, the treatment coverage rose from the original 40.3% at baseline, through 68.7% in 2011 to 74.1% in 2012 (VCD, Unpublished records). Unfortunately, the GNNTD support was temporary and in order to improve on this level of treatment coverage, more sustainable collaborations must be created.

Intersectoral collaboration [23] between health and fisheries department could be play a major role in improving the treatment coverage. Most of the migratory fishing communities who often miss treatment are persistently in contact with fisheries officers. Hence if the fisheries department was involved in drug distribution, high treatment coverage would always be assured.

Furthermore, earlier parasitological data collected in 2010 [18] indicated that co-infections of malaria with more than one helminth was often the norm rather than the exception in islands where schistosomiasis, STH and Lymphatic filariasis are endemic. This scenario calls for an integrated approach to control helminthiasis and malaria. Although earlier co-infections studies yielded conflicting results; with some suggesting that malaria leads to increased susceptibility to *S. mansoni* infection [24, 25], while others reported modulation of malaria via up-regulation of the immune system as a result of helminth infection [26], nevertheless, both infections are harmful and highly prevalent necessitating urgent attention. This would imply that several drugs and other interventions are administered in increased collaboration between the relevant programmes to maximize cost effectiveness and to promote sustainability of disease control efforts [18].

It was encouraging to hear from the community that they were comfortable to receive treatment and that they felt better following the treatment. This implies that if there is adequate support, treatment compliance is still high in the islands and there is potential for improved treatment coverage. Community participation is considered to be a key approach towards controlling NTDs since it increases community autonomy and programme ownership [23]. In 2012 MDA, 1312 islanders including CMDs, supervisors and health workers were trained with the hope that if many local people have skills to contribute to programme implementation, naturally treatment coverage would increase. Indeed the programme achieved increased treatment during that period from 68.7% in 2011 to 74.1% in 2012. (VCD, Unpublished records). Hence continuous effort should be made to empower island community members to participate in drug distribution and to adhere to taking their drugs.

Results from this study have clearly demonstrated that there are many other challenges that must be overcome to achieve effective disease control in the islands. Generally, health facilities are few, understaffed, most biomedical staffs have no formal training, supply of praziquantel in health facilities is inadequate and there is severe shortage of technicians and diagnostic equipment. For example, while it was reported that many clients complained of diarrhea and abdominal pain, very few biomedical staff could associate these symptoms to schistosomiasis and yet most of them had claimed they relied on symptoms to identify schistosomiasis cases.

These challenges coupled with discouraging attitude of healthcare staff thwart good case management. In addition, the healthcare staff seems to be over burdened with other illnesses such as malaria and HIV, which limit their participation in NTD MDAs. All these factors imply that facility based schistosomiasis management in islands is very poor. It is vital to improve skills of healthcare staff so that if anyone with suspicious symptoms is identified, that person is promptly treated. Asked where they would go for treatment if they had schistosomiasis, most of the respondents claimed that they would seek for remedy from biomedical health workers.

**Figure 4:**
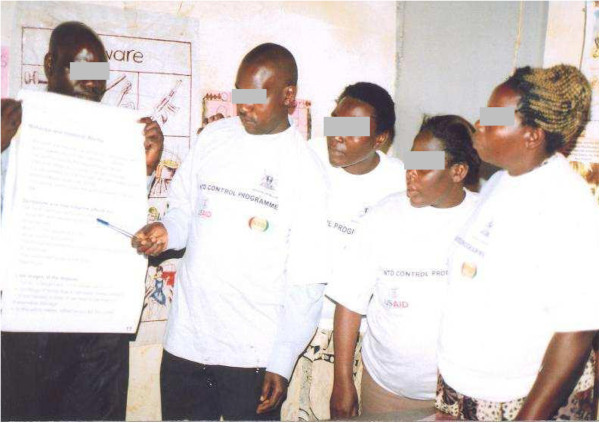
**To show that those Drug Distributors who were trained were each offered a T-shirt by RTI- ENVISION.**

However, they had a few concerns including long and costly distance to health facilities, lack of transport funds and demand for bribes by healthcare workers. Since most treatment for schistosomiasis is mainly offered through MDAs, the major threat to the programme is sustained voluntarism of drug distributors. Usually CMDs start dropping out of service as soon as they realize that they will not be paid for their work [27]. However, in this study, it was encouraging to note that CMDs were willing to continue distributing treatments even though they were not paid. During the 2012 MDA, NTD/RTI provided a T-shirt to each drug distributor (Figure 4) which further encouraged their resolve to continue drug distribution.

Data from this study has indicated that awareness and knowledge about schistosomiasis is low even among biomedical health staff and many negative misconceptions exist especially in regard to disease transmission and prevention. Most people including biomedical staff do not know schistosomiasis signs and symptoms indicating that previous health education messages were either inappropriate or poorly delivered. Majority of respondents indicated that drinking un-boiled water was responsible for bilharzia transmission; a wrong belief strongly demonstrating that there is a critical need for targeting health education messages both to health workers and the communities. As long as the at risk population is not adequately informed about the risky behavior, they will continue to spread the infection [28].

Regrettably, designing of effective behavior change messages in a setting where there is serious lack of attractive alternatives such as safe water supply and good sanitary facilities is unrealistic. Most often, health education messages are conveyed in a way that appears irrelevant to local experience [29]. The current data indicated that most people knew the health hazards that accrue from their unsanitary behavior but they had no control of this situation. Only one school had a functional hand washing facility. Most people were aware that they got schistosomiasis through contact with Lake Water but in islands where fishing is the main economic activity, contact with lake water is inevitable. While up to 90.8% of the respondents preferred to use a latrine especially for disease prevention, only 33.8% of the households had a latrine; most of them filthy, 10.5% with no doors and 13.7% dilapidated implying that most of them were not convenient for use [30]. Consequently, up to 66.2% of the islanders practiced open air defecation (Figure 3).

Our data showed that land on islands belong to a few land lords who own most of the huts. Majority of the inhabitants are mere tenants with no space or authority to construct a latrine. Even then, it is costly for individuals to construct latrines due to sandy soils. The construction requires expensive materials which most islanders cannot afford and without such materials, the latrines would either be swept away by floods or sink after a very short period. Thus lack of this fundamental sanitary facility, the latrine, rather than refusal to use it or ignorance is the main problem.

Our data showed that island inhabitants have some knowledge about schistosomiasis mainly from school or biomedical staff. Only 5% of their knowledge is from radio/TV talk shows, jingles and IEC materials (1.8%) on which the programme spends most of the funds. Perhaps poor socio-economic status of the rural people where only a few of them own either a radio or TV set could be the main barrier of media information flow. Their knowledge increased with increasing length of residence and with increasing educational attainment.

It had earlier been believed that school enrolment rate in fishing villages in Uganda is very low (Kabatereine et al. [21]), However, our results revealed that the enrolment rate was about 83%. Thus school health education can be very effective in the islands reinforcing community messages through health workers and CMDs. Messages given to large numbers of school children are known to quickly spread beyond school to the community particularly to mothers [31, 32]; hence school health education should be strengthened.

The ultimate goal is the elimination of schistosomiasis. In 2012, World Health Assembly Resolution 65.19 recommended that countries, if possible, should aim beyond control of morbidity towards elimination of the disease [33]. This was a bold step perhaps based on the pledge by pharmaceutical companies to donate sufficient drugs. To achieve elimination will necessitate high and consistent treatment coverage and in high transmission areas, this will entail inclusion of preschool age children in preventive chemotherapy efforts [34].

Elimination will also necessitate committed investments in preventive measures because even though preventive chemotherapy can significantly reduce pathology, it cannot interrupt transmission in a focus where transmission is intense [35]. Hence access to and use of clean water and improved sanitation are essential in achieving elimination of schistosomiasis [36]. This approach would also aid in combating numerous other pathogens that are transmitted via the faecal-oral route [1].

Unfortunately, in these islands, socio-economic development is slow, sanitation appalling and there is no clean water. In such a setting, schistosomiasis transmission will remain high such that if treatment ceases, prevalence will quickly return to the pre-treatment levels [19, 20]. Unfortunately, improvement in water and sanitation are usually slow and often go hand in hand with a general increase in economic development [37]. In most schistosomiasis endemic areas, these services are usually inadequately provided perhaps due to competing necessities [1]. The nasty fact is that without these developments, elimination of schistosomiasis will remain a distant goal [38]. Under the current situation, rather than schistosomiasis elimination [33], morbidity control will remain the main target far beyond the 2020s in the islands.

## Conclusion

These investigations have fulfilled a huge gap of providing KAP data in the Lake Victoria islands. Fortunately, the study was performed before designing a Ugandan NTD communication strategy for behavior change. Our results therefore are invaluable in the designing of feasible health education messages targeted to raising community awareness on schistosomiasis. The data will also be utilized as a benchmark for monitoring impact of the interventions in the islands. Rather than elimination, our results indicate that the programme will continue to target morbidity control until preventive measures have been improved. Access to safe water is one of the important positive steps towards schistosomiasis control but it will be long before provision of this asset is achieved. As a short term solution, it is advisable to consider other feasible alternatives such as empowerment of local communities with skills to harvest and store rain water. For sanitation, it is important to promote latrine use through vigorous health education messages and to urge island landlords to ensure that all their rented houses muss possess a latrine. Finally, the government should equip health facilities with schistosomiasis diagnostic tools, provide adequate trained staff and stock praziquantel in all health facilities in the endemic areas.
